# Supplementary material for: Genetic and neuronal regulation of sleep by neuropeptide VF
Source: eLife. 2017 Nov 6;6:e25727. doi: 10.7554/eLife.25727 (PMC5705210; doi:10.7554/eLife.25727)
Supplement: Transparent reporting form [file elife-25727-transrepform.pdf]

eLIFE
1st Floor
24 Hills Road
Cambridge CB2 1JP, UK
P 01223 855340
W elifesciences.org
T @elife

eLife's transparent reporting form

We encourage authors to provide detailed information *within their submission* to facilitate the interpretation and replication of experiments. If you have any questions, please contact us: editorial@elifesciences.org.

Sample-size estimation

- You should state whether an appropriate sample size was computed when the study was being designed
- You should state the statistical method of sample size computation and any required assumptions
- If no explicit power analysis was used, you should describe how you decided what sample (replicate) size (number) to use

Please outline where this information can be found within the submission (e.g., page numbers or figure legends), or explain why this information doesn't apply to your submission:

No explicit power analysis was used. However, behavioral experiments were performed with large sample sizes (N>30 per group per experiment) in order to obtain representative samples of the population. Three or more independent replicate experiments were performed for nearly all behavioral experiments, unless otherwise stated in figure legends.

Replicates

- You should report how often each experiment was performed
- You should include a definition of biological versus technical replication
- The data obtained should be provided and sufficient information should be provided to indicate the number of independent biological and/or technical replicates
- If you encountered any outliers, you should describe how these were handled
- Criteria for exclusion/inclusion of data should be clearly stated
- High-throughput sequence data should be uploaded before submission, with a private link for reviewers provided (these are available from both GEO and ArrayExpress)

Please outline where this information can be found within the submission (e.g., page numbers or figure legends), or explain why this information doesn't apply to your submission:

The data presented provides sufficient information to indicate the number of independent biological and/or technical replicates. This data is displayed in the figures legends. Criteria for exclusion/inclusion of data are clearly stated in the Material and Methods.

eLife Sciences Publications, Ltd is a limited liability non-profit non-stock corporation incorporated in the State of Delaware, USA, with company number 5030732, and is registered in the UK with company number FC030576 and branch number BR015634 at the address 1st Floor, 24 Hills Road, Cambridge CB2 1JP | August 2014

1st Floor
24 Hills Road
Cambridge CB2 1JP, UK

P 01223 855340
W elifesciences.org
T @elife

Statistical reporting

- Statistical analysis methods should be described and justified
- Raw data should be presented in figures whenever informative to do so (typically when N per group is less than 10)
- For each experiment, you should identify the statistical tests used, exact values of N, definitions of center, methods of multiple test correction, and dispersion and precision measures (e.g., mean, median, SD, SEM, confidence intervals; and, for the major substantive results, a measure of effect size (e.g., Pearson's r, Cohen's d)
- Report exact p-values wherever possible alongside the summary statistics and 95% confidence intervals. These should be reported for all key questions and not only when the p-value is less than 0.05.

Please outline where this information can be found within the submission (e.g., page numbers or figure legends), or explain why this information doesn't apply to your submission:

Statistical analysis methods are clearly described and justified in the Material and Methods section. The statistical methodology used is described in the Figure legends. Raw data (where N per group is less than 10) is presented in figures.

(For large datasets, or papers with a very large number of statistical tests, you may upload a single table file with tests, Ns, etc., with reference to page numbers in the manuscript.)

Additional data files ("source data")

- We encourage you to upload relevant additional data files, such as numerical data that are represented as a graph in a figure, or as a summary table
- Where provided, these should be in the most useful format, and they can be uploaded as "Source data" files linked to a main figure or table
- Include model definition files including the full list of parameters used
- Include code used for data analysis (e.g., R, MatLab)
- Avoid stating that data files are "available upon request"

Please indicate the figures or tables for which source data files have been provided:

Behavioral data presented in the manuscript utilized custom written software code, and has been included as source data.

Scripts used for analysis of behavioral data. *sort_fish_sttime_192.pl* is a Perl script (Prober et al., 2006) that converts data acquired by the Viewpoint videotracker system to a format that is useful for analysis using Matlab and removes notations that are not relevant to behavioral analysis. *perl_batch_192well.m* is a Matlab script

that allows the *sort_fish_sttime_192.pl* script to run on the Matlab platform. *TapAnalysis.m* is a Matlab script that analyzes tapping assay data and generates a table that lists the number of larvae that moved during each tapping event. *VT_analysis.m* is a Matlab script (modified from Prober et al., 2006) that analyzes locomotion data collected by the Viewpoint videotracker system to quantify several metrics, including activity, waking activity, sleep, sleep architecture and sleep latency. Detailed instructions on the use of these scripts will be provided upon request.

eLife Sciences Publications, Ltd is a limited liability non-profit non-stock corporation incorporated in the State of Delaware, USA, with company number 5030732, and is registered in the UK with company number FC030576 and branch number BR015634 at the address 1st Floor, 24 Hills Road, Cambridge CB2 1JP | August 2014
